# Hemoglobin A1c Levels Modify Associations between Dietary Acid Load and Breast Cancer Recurrence

**DOI:** 10.3390/nu12020578

**Published:** 2020-02-23

**Authors:** Tianying Wu, Fang-Chi Hsu, Shunran Wang, David Luong, John P. Pierce

**Affiliations:** 1 Division of Epidemiology and Biostatistics, School of Public Health, San Diego State University, San Diego, CA 92182, USA; luongdavid23@gmail.com; 2 Morres Cancer Center, University of California at San Diego School of Medicine, San Diego, CA 92093, USA; jppierce@ucsd.edu; 3 Department of Biostatistics and Data Science, Division of Public Health Sciences, Wake Forest School of Medicine, Winston-Salem, NC 27157, USA; fhsu@wakehealth.edu; 4 Department of Mathematics and Statistics, College of Science, San Diego State University, San Diego, CA 92182, USA; swang7@sdsu.edu

**Keywords:** Dietary acid load, cancer survivors, cancer recurrence

## Abstract

Background: Metabolic acidosis promotes cancer metastasis. No prospective studies have examined the association between dietary acid load and breast cancer recurrence among breast cancer survivors, who are susceptible to metabolic acidosis. Hyperglycemia promotes cancer progression and acid formation; however, researchers have not examined whether hyperglycemia can modify the association between dietary acid load and breast cancer recurrence. Methods: We studied 3081 early-stage breast cancer survivors enrolled in the Women’s Healthy Eating and Living study who provided dietary information through 24-h recalls at baseline and during follow-up and had measurements of hemoglobin A1c (HbA1c) at baseline. We assessed dietary acid load using two common dietary acid load scores, potential renal acid load (PRAL) score and net endogenous acid production (NEAP) score. Results: After an average of 7.3 years of follow-up, dietary acid load was positively associated with recurrence when baseline HbA1c levels were ≥ 5.6% (median level) and ≥5.7% (pre-diabetic cut-point). In the stratum with HbA1c ≥ 5.6%, comparing the highest to the lowest quartile of dietary acid load, the multivariable-adjusted hazard ratio was 2.15 (95% confidence interval [CI] 1.34-3.48) for PRAL and was 2.31 (95% CI 1.42-3.74) for NEAP. No associations were observed in the stratum with HbA1c levels were <5.6%. P-values for interactions were 0.01 for PRAL and 0.05 for NEAP. Conclusions: Our study demonstrated for the first time that even at or above normal to high HbA1c levels, dietary acid load was associated with increased risk of breast cancer recurrence among breast cancer survivors. Impacts: Our study provides strong evidence for developing specific dietary acid load guidelines based on HbA1c levels.

## 1. Introduction

Breast cancer survivors are susceptible to recurrence. Analyses based on Surveillance, Epidemiology, and End Results (SEER) reported that approximately 36.8% of breast cancer survivors had recurrence within 10 years, with most of these recurrences (81.9%) occurring within 5 years after diagnosis [1]. Dietary and lifestyle factors play an important role in breast cancer recurrence among breast cancer survivors; however, general dietary guidelines are not specifically tailored to breast cancer survivors. Dietary patterns such as Western dietary patterns, prudent dietary patterns, and the alternative health index, and their associations with breast cancer mortality have been assessed in previous cohort studies among cancer survivors, but the results were not consistent [2]. Results related to the consumption of specific types of foods, such as red meat [3] or total fruits and vegetables [4], were either not significant or weakly associated. One important reason for these inconsistent results may be the fact that people respond to food differently. For instance, glycemic responses were dramatically different based on different microbiome profiles across individuals [5]. In addition, some studies have focused on modifications by gene [6,7] or smoking status [8]. Clinical biomarkers not only reflect the endogenous biological status, but are also commonly used in clinics; if they can be used as one method to identify subgroups susceptible to certain types of diets, they will help make recommendations for individualized nutrition for breast cancer survivors. Individualized nutrition is an emerging trend, yet this type of research is limited among breast cancer survivors.

Although metabolic acidosis can promote cancer metastasis [9,10,11,12], dietary acid load may contribute to metabolic acidosis if the acid–base balance is not properly adjusted. Cancer patients have a reduced capacity to adjust their acid–base balance [13,14]. Therefore, it is important to assess whether dietary acid load is associated with breast cancer recurrence. Western diets, consisting of lower fruit and vegetable intakes and higher meat consumption, are considered to be acid-producing diets. A recent publication from our group reported that dietary acid load was associated with hyperglycemia among breast cancer survivors [15]. Elevated blood glucose was proposed to be associated with tumorigenesis decades ago [16]. Hyperglycemia has been found to be associated with an increased risk of developing cancer in cohort studies [17]. Women with breast cancer are also more likely to develop diabetes than women without breast cancer [18]. Furthermore, high glucose levels can promote an acid environment by forming lactate during glycolysis [19]. However, researchers have never examined whether hyperglycemia can modify the impact of dietary acid load on cancer recurrence.

We will leverage a large cohort, the Women’s Healthy Eating and Living (WHEL) study, to conduct the current study. Because this cohort was originally a dietary trial of increasing vegetable and fruit intakes and reducing fat intakes, the range of dietary acid load is wider in this cohort than the typical American diet, which enabled us to better examine the dose–response relationship of dietary acid load with breast cancer recurrence.

This study aimed to determine whether dietary acid load is a risk factor of breast cancer recurrence among early-stage breast cancer survivors and whether hemoglobin A1c (HbA1c), a biomarker reflecting glucose status in the past three months, can modify this association. We hypothesized that dietary acid load would be positively associated with the incidence of breast cancer recurrence and the association would be stronger in women with high levels of HbA1c.

## 2. Materials and Methods

### 2.1. Study Design

This study is a prospective cohort, the Women’s Healthy Eating and Living (WHEL) study, comprising mainly early-stage (stage I, II, or IIIA) breast cancer survivors. Between 1995 and 2000, the WHEL study enrolled 3088 women within 4 years of diagnosis. The WHEL study was initially a multi-site randomized trial designed to test whether a diet low in fat and rich in vegetables, fruit, and fiber reduced the risk of invasive breast cancer recurrence and death from any cause in women. Briefly, inclusion criteria were as follows: women who had stage I (≥1 cm), II, or IIIA breast cancer diagnosed within the previous 4 years, had no evidence of cancer recurrence, had completed primary therapy, were 18–70 years old at diagnosis, did not have life-threatening comorbidities, and were able to communicate dietary data via 24-h food recall. Exclusions included insulin dependence and the diagnosis of a comorbidity requiring a specific diet or the use of a medication that contraindicated a high-fiber diet and insulin dependence. Women with diagnoses after age 70 years and those with stage 1 tumors smaller than 1 cm were also excluded. Extensive details can be found in previous publications from the WHEL study [20,21]. Briefly, two thirds of these women were under 55 years of age at randomization, and participants were followed through to 2006. The intervention did not significantly change breast cancer prognosis after an average of 7.3 years of follow-up. Therefore, the present study considers and analyzes the study sample as a single cohort while controlling the initial trial assignment. After removing participants who did not have baseline dietary record or measurement of plasma HbA1c, we included 3081 participants in this cohort.

The Institutional Review Board at the University of California at San Diego approved the original study. All subjects provided written informed consent. The de-identified data were provided by the principal investigator of the WHEL study. The current study was an ancillary study using the de-identified data from the WHEL study, thus the exempt IRB was approved by the San Diego State University IRB committee (protocol number: Temp-1286).

### 2.2. Dietary Assessment

At baseline, Year 1, and Year 4, dietary intake was assessed by four prescheduled, 24-h dietary recalls collected by telephone on random days over a 3-week period: two on the weekends and two during weekdays. Dietary assessors used the multi-pass software-driven recall protocol of the Nutritional Data System software (NDS-R, 1994-2006, 91 University of Minnesota, Minneapolis, MN, USA).

In terms of the assessment of dietary acid load, two commonly used scores were used to estimate dietary acid load in epidemiological studies: the potential renal acid load (PRAL) score and the net endogenous acid production (NEAP) score. The PRAL score considers the intestinal absorption rates for contributing nutrient ionic balances for protein, potassium, calcium, and magnesium and the dissociation of phosphate at pH 7.4 [22]. Frassetto et al. [23] developed the NEAP score, which uses total protein and potassium intake as the main components involved in acid production. PRAL and NEAP scores were derived from estimations of several nutrient intakes, as follows [24]:

PRAL (mEq/day) = (0.49 × protein [g/day]) + (0.037 × phosphorus [mg/day]) − (0.021 × potassium [mg/day]) − (0.026 × magnesium [mg/day]) − (0.013 × calcium [mg/day])

NEAP (mEq/day) = (54.5 × protein [g/day]/potassium [mEq/day]) − 10.2

This study used both scores for dietary acid assessment because they reflect slightly different nutritional intakes and biological mechanisms. A Western diet rich in animal products is an acid-producing diet whereas a diet rich in fruit and vegetables is an alkaline-producing diet. A negative PRAL value reflects an alkaline-forming potential; a positive value reflects an acid-forming potential [25]. For NEAP, there is large variation in the general population (ranging from 10 to 150 mEq/day), although a typical Western diet has been characterized by a NEAP score of approximately 50 mEq/day [23,26].

### 2.3. Assessment of Study Outcome

The primary outcome of this study is breast cancer recurrence. Information on outcomes were obtained through health status questionnaires completed during each of five clinic visits scheduled during the 6 years as well as during semiannual telephone interviews. Any reported event/death led to a medical record/death certificate review by two independent study physicians. In addition, participants out of study contact were searched for end points using the National Death Index (NDI). Breast cancer recurrence was defined as the combination outcome of invasive breast cancer recurrence (local, regional, or distal) or new primary breast cancer. Carcinoma in situ was not counted as a study outcome. Approximately 4% of study participants were lost to follow-up, and these were censored at date of last contact. At the close of the study in June 2006, vital status was known for 96% of the participants. Mortality data were updated through September 2009 using the Social Security Death Index. Median follow-up time was 7.3 years for breast cancer event-free survival. For participants without an event, follow-up time was censored at the time of the participant’s death, at the last documented staff contact date, or at the study completion date (June 1, 2006).

### 2.4. Measurement of HbA1c 

HbA1c was measured using ion exchange high-performance liquid chromatography (D-10 system; Bio-rad laboratories, Hercules, CA, USA) in washed red blood cells collected at the baseline visit. The coefficients of variation were 1.5% and 1.6%, respectively, for high- and low-quality controls.

### 2.5. Other Assessments

Demographic characteristics were self-reported. Variables abstracted from patient records included initial cancer diagnosis and treatment. Specific variables abstracted included tumor stage, size, hormone receptor status, and use of radiation, chemotherapy, and/or post treatment anti-estrogens use. Physical activity levels were assessed using an adapted validated questionnaire from the Women’s Health Initiative [27]. Physical activity was converted into metabolic equivalent tasks (METs), as previous studies did [28].

### 2.6. Statistical Analyses 

We used Cox proportional hazard models to assess the association between dietary acid load and incidence of breast cancer recurrence. Time was calculated from the study entry to the time when participants were diagnosed with the incidence of recurrent breast cancer, died, were lost to follow-up, or censored at the end of follow-up period, whichever came first. As previously introduced, dietary acid load was characterized by PRAL and NEAP scores. Repeated measures of PRAL and NEAP at Year 0, Year 1, and Year 4 were analyzed by modeling them as time-varying covariates using the Cox proportional hazard regression. PRAL and NEAP scores were classified into quartiles or tertiles using the average intakes at years 0, 1, and 4 to set up the cut-point for each quartile or tertile. The multivariable Cox regression models were used to estimate hazard ratios. Death was considered as a competing risk. We controlled the following covariates based on a priori assumption: age at diagnosis, race/ethnicity, education level, intervention group, menopausal status at baseline, total calorie intake, alcohol intake, smoking status, pack-years, physical activity, body mass index (BMI), education level, tumor stage, tumor size, estrogen and progesterone receptor status, type of anti-estrogens, radiotherapy, and chemotherapy. Among these covariates, time-varying covariates included BMI, physical activity, smoking status, pack-years, total calorie intake, alcohol intake, and types of anti-estrogen therapy. We controlled these covariates in all the multivariable models, except for analyses conducted in four HbA1c strata (presented in Table 1). Due to smaller sample sizes in four strata by HbA1c levels, we only controlled covariates or time-varying covariates that changed the hazard ratios by more than 10%.

Because we hypothesized that HbA1c can modify the association between dietary acid load and risk of breast cancer recurrence, the interaction terms between dietary acid load and categorized hemoglobin A1c levels were additionally included in the models. Hemoglobin A1c levels were categorized by quartiles (<5.3%, 5.3–5.6%, 5.6–5.7% and ≥5.7%), by median (<5.6 and ≥5.6%) and by pre-diabetic range (<5.7% and ≥5.7%). If interactions were significant, meaning the effect of dietary acid load was different among hemoglobin A1c groups, then the stratified analysis were performed. Cox models were used to determine these associations in stratified analyses. To assess whether a significant interaction occurred between dietary acid load and HbA1c, we used the Wald P-value for the interaction term in a model that also included the main effects. 

The proportional hazards assumption was examined and satisfied in all Cox proportional hazards regression models by testing the significance of the product terms for our variable of interest and log time. All analyses were conducted using SAS version 9.4 (SAS Institute, Cary, NC, USA).

## 3. Results

### 3.1. Baseline Characteristics by Quartiles of PRAL Score in the Entire Cohort 

The correlation between PRAL and NEAP is 0.96; therefore, we only presented baseline characteristics according to PRAL. As shown in Table 2, compared to women with a low dietary acid load, women with higher dietary acid load were younger, included higher proportions of obese and overweight women, and were likely to have lower education and physical activity. We also examined the nutrients that are elements used for calculating PRAL or NEAP. A high PRAL diet was associated with higher fat and protein intakes but lower carbohydrate intakes. It was also characterized by lower intakes of calcium, potassium, and magnesium.

### 3.2. Baseline Characteristics by Quartiles of PRAL Score in Different HbA1c Strata

We used the median of HbA1c at 5.6% to classify two groups: low-HbA1c group (<5.6%) and high-HbA1c group (≥5.6%). As shown in Table 3, the age ranges tended to be younger in the low-HbA1c group than the high-HbA1c group, but age appeared to be inversely associated with PRAL in both groups. The trend of the associations of demographic covariates with PRAL were more or less similar between the low-HbA1c and high-HbA1c groups except for a few variables—namely, normal weight, education, and physical activities. With the increase of PRAL from quartile 1 to quartile 4, the percent of normal weight decreased by 34% in the low-HbA1c group but by 58% in the high-HbA1c group; the percent of education at or above college level decreased by 34% in the low-HbA1c group but by 43% in the high-HbA1c group; and the medians of physical activities decreased by 36% in the low-HbA1c group but by 70% in the high-HbA1c group. For nutrients, the trends of associations of these nutrients with changes of PRAL were somewhat similar, although the overall levels of these nutrients between the high-HbA1c and low-HbA1c groups were different, as were the magnitudes of the changes of several nutrients with changes of PRAL. For instance, total energy, carbohydrate, calcium, phosphorus, potassium, and magnesium intakes tended to higher in the low-HbA1c group than in the high-HbA1c group. Total fat intakes tended to be lower in the low-HbA1c group than the high-HbA1c group. With the increases of PRAL from quartile 1 to quartile 4, the reduction of carbohydrates was 23% in the low-HbA1c group but only 19% in the high-HbA1c group; the increase of fat intake was 29% in the low-HbA1c group but 36% in the high-HbA1c group; the percent of protein increase was 16% in the low-HbA1c group but 10% in the high-HbA1c group; and the potassium reduction was 43% in the low-HbA1c group but 27% in the high-HbA1c group.

### 3.3. Dietary Acid Load and Risk of Breast Cancer Recurrence

During the average of 7.3 years of follow-up, 517 validated recurrent cases in total were confirmed. These included 357 distant recurrent cases. Neither PRAL nor NEAP was associated with an increased risk of incident breast cancer recurrence in age- or multivariable-adjusted models. As shown in Table 1, the multivariable-adjusted HRs between extreme quartiles were 1.16 (95%CI, 0.89-1.50) for PRAL and 1.19 (95%CI, 0.91-1.55) for NEAP; *p* values for the trends were > 0.2 for both PRAL and NEAL. When we considered only distant recurrent cases, the results were similar, and no significant associations were found between dietary acid load and distant recurrence for two acid load scores.

### 3.4. Stratification by Hemoglobin HbA1c Levels

To determine whether HbA1c levels can modify the association between dietary acid load and breast cancer recurrence, we first created four strata using the cut-point of the quartile range of HbA1c. As shown in Table 4, we observed positive associations between dietary acid load and breast cancer recurrence in two strata with HbA1c ≥ 5.6%, whereas null associations emerged in two strata with HbA1c < 5.6%. *P* values for interactions were ≤0.05 for both PRAL and NEAP. Thus, we further collapsed the two strata with HbA1c < 5.6% into one stratum and two strata with HbA1c ≥ 5.6% into one stratum. No associations were found in stratum with HbA1c < 5.6% but positive associations were found in stratum with HbA1c ≥ 5.6%. As shown in Table 5, in the stratum with HbA1c ≥ 5.6%, both PRAL and NEAP scores were associated with an increased risk of breast cancer recurrence in age-adjusted and multivariable-adjusted models. In multivariable-adjusted analyses, there were a statistically significant linear trend of dietary acid load scores with breast cancer recurrence (*p* for trend = 0.0005 for PRAL and *p* for trend = 0.0004 for NEAP). Women with PRAL at quartile 4 (PRAL ≥ 7 mEq/day) showed 2.15 (95% CI 1.34-3.48) times the risk of breast cancer recurrence than women with PRAL at quartile 1 (PRAL < -19.5 mEq/day). A greater magnitude was observed for the NEAP score. The HR between extreme quartiles of NEAP was 2.31 (95% CI, 1.42-3.74). No associations were observed for strata with HbA1c < 5.6%. Further, we removed local and regional cases and analyzed the association of acid load with distant recurrent cases in the stratum with HbA1c <5.6% and the stratum with HbA1c ≥ 5.6%. The associations were similar, with the exception of NEAP, where the association was stronger in the HbA1c ≥ 5.6% stratum. The HR comparing the highest to the lowest quartile of NEAP was 2.48 (95% CI, 1.36-4.51).

Moreover, we conducted additional analyses in the stratum with HbA1c ≥ 5.6%. We simultaneously analyzed the PRAL with acid-contributing foods, including fresh red meat, processed meat, cruciferous vegetables, allium vegetables, legumes, soy legumes, and other vegetables (Appendix A Table A1). We found that the associations between dietary acid load remained significant, although attenuated by 9%, whereas many of the foods included were not significantly associated with total recurrence. One exception was cruciferous vegetables and legumes, which were modestly and inversely associated with breast cancer recurrence with or without adjustment of PRAL. In the model adjusted with PRAL, the HR between extreme quartiles was 0.82 (95% CI 0.48-1.40) for cruciferous vegetables and 0.66 (95% CI 0.44-0.99) for legumes; *p* values for trends were 0.02 for both.

Similar patterns were found when we used the pre-diabetic range to create two strata, one stratum with HbA1c < 5.7% and one stratum with HbA1c ≥ 5.7%. Positive associations were observed in stratum with HbA1c ≥ 5.7% but no associations in stratum with HbA1c < 5.7% (data not shown).

## 4. Discussion

In this prospective cohort of early-stage breast cancer women in the USA, a diet with a high acid load, characterized by a high PRAL or high NEAP score, was associated with an increased risk of incidence of breast cancer recurrence among women at or above normal to high levels of HbA1c (HbA1c ≥ 5.6%). Among this group, a high-acid load diet can increase breast cancer recurrence by at least 2 times compared to a low-acid load diet. No associations were found between dietary acid load and breast cancer recurrence in the low HbA1c group (<5.6%). Our findings provide important messages for clinicians and clinical dieticians that we should pay attention to both dietary acid load and HbA1c levels; the cut-point for HbA1c should be lower than the pre-diabetic cut-point (5.7%) when considering the joint impact of dietary acid load and HbA1c.

It is not surprising that we found positive and significant associations among women with higher HbA1c levels but a null association between dietary acid load and breast cancer recurrence in women with low HbA1c levels. We postulate that the following reasons may explain our findings. Dietary acid load and hyperglycemia may have synergistic impacts on breast cancer progression. Dietary acid load can promote metabolic acidosis if acid–base balance cannot be appropriately adjusted. Cancer patients have a reduced capacity to adjust acid–base balance [13,14]. Metabolic acidosis has been shown to stimulate cancer metastasis in cell and animal models [9,10,11,12]. Dietary acid load plays an important role in the development of type 2 diabetes [29], characterized by hyperglycemia status. We also previously demonstrated that dietary acid load was associated with increased HbA1c among breast cancer survivors [15]. Glucose has been shown to be a primary driving force for the growth of tumor cells for more than two decades [30], and glucose can promote acidosis through the formation of lactic acid during glycolysis [19]. Furthermore, hyperglycemia often occurs in tandem with hyperinsulinemia in the pre-diabetic stage [31]. Hyperinsulinemia is a risk factor for breast cancer progression [32,33,34]. Insulin can also stimulate glycolysis, which often causes lactic acid production and increased concentrations of ketone bodies [31]. Moreover, acidic environments can also decrease glucose uptake [35,36], which can further worsen the hyperglycemia status and promote cancer progression. Prospective studies have shown that HbA1c was associated with increased total breast cancer incidence [37] and stage II–IV breast cancer incidence [38]. Few studies have investigated the associations of HbA1c with recurrence among breast cancer survivors. A previous publication from the WHEL cohort observed a positive association between HbA1c and total mortality, but only among breast cancer survivors whose HbA1c ≥ 6.5%, which is a diabetic range [39]. Our study demonstrated synergistic impacts of dietary acid load and hyperglycemia on breast cancer recurrence, as the cut-point of HbA1c was much lower (≥5.6%) when we considered dietary acid load and HbA1c together than when considering HbA1c only (≥6.5%), as a previous study did [39]. Finally, inflammation plays an important role in cancer progression [40]. We previously demonstrated that dietary acid load can increase inflammation among breast cancer survivors [15]. Thus, dietary acid load could also promote breast cancer recurrence through inflammation.

Our study also demonstrated that dietary acid load can better capture the acid–base influences than some acid-contributing (e.g., red meat) or alkaline-contributing foods (e.g., different types of vegetables) and was associated with breast cancer recurrence independent of the acid- or alkaline-contributing foods, as shown in Table A1 in Appendix A. Although the associations were somewhat attenuated after the adjustment of acid- or alkaline-contributing foods, such a reduction is reasonable as these foods are correlated with dietary acid load scores. Significant associations after the adjustment of these foods further assure us that dietary acid load was independently associated with breast cancer recurrence among breast cancer survivors and was not just a surrogate marker for evaluating the combined consumption of red meat and vegetables.

This study has several strengths. This is the first large prospective cohort study investigating the associations of dietary acid load with breast cancer recurrence among breast cancer survivors. Four 24-h recalls during each visit were the unique advantages of this cohort, but were rarely conducted in other cohorts. The repeated measurements of dietary assessment at baseline, Year 1, and Year 4 enabled us to assess the longitudinal impact of dietary acid on breast cancer recurrence. As this study was originally a trial of high-vegetable, high-fruit, and low-fat intake interventions, we observed a wider range of dietary acid load than other cohorts and an immediate shift of dietary acid load to lower levels from baseline to year 1. This finding enabled us to better assess a longitudinal dose–response relationship of dietary acid load with the outcome. This study was also the first large prospective study among breast cancer survivors to assess the associations between dietary acid load and breast cancer recurrence; the large sample size provided us with sufficient power and enabled us to adjust for multiple covariates.

This study also faced several limitations. First, the original randomization was not based on dietary acid load, thus we analyzed the data as observational data. Although causal-effect conclusions cannot be obtained, cohort analyses provide the strongest evidence for making causal suggestions among different types of observational studies and provide strong evidence for conducting future clinical trials. Using HbA1c levels to predict group responses to dietary acid load in a clinical trial will likely be a new emerging type of clinical trial—namely, an adaptive clinical trial targeting individualized nutrition. Furthermore, the average follow-up was 7.3 years, not 20 years; thus, the long-term impact of dietary acid load on breast cancer recurrence could not be determined in this study.

## 5. Conclusions.

Our study further highlights that it is important to reduce a high-acid load diet for breast cancer survivors who had normal to high (≥5.6%) or moderate to high level of abnormal range of HbA1c (≥5.7%). To our knowledge, this is the first study to show that dietary acid load is a risk factor for breast cancer recurrence among women who have moderately increased long-term glucose levels, as measured by HbA1c. Our findings suggest a significant interaction between dietary acid load and HbA1c. When considering dietary acid load together with HbA1c, the cut-point for increased risk should be lower than the pre-diabetic cut-point of 5.6%. Our study provided strong evidence for making tailored dietary guidelines in terms of dietary acid based on HbA1c status among breast cancer survivors.

## Figures and Tables

**Table 1:** Dietary acid load and risk of breast cancer recurrence in entire WHEL cohort.

			HR (95% CI)	
	**PRAL (mEq/day)**			
	Range	No. events/person-years	Age-adjusted	Multivariable-adjusted
Quartile 1	<-19.50	62/3149	1	1
Quartile 2	-19.50 to <-6.94	139/5590	0.99 (0.78–1.28)	1.06 (0.83–1.36)
Quartile 3	-6.94 to <3.22	153/6319	1.10 (0.86–1.41)	1.12 (0.87–1.44)
Quartile 4	≥3.22	163/5925	1.16 (0.91–1.48)	1.16 (0.89–1.50)
*p* for trend			0.19	0.41
	NEAP (mEq/day)			
	Range	No. events/person-years	Age-adjusted	Multivariable-adjusted
Quartile 1	<28.44	63/3233	1	1
Quartile 2	28.44 to <37.25	131/5601	1.10 (0.86–1.41)	1.08 (0.84–1.40)
Quartile 3	37.25 to <46.90	160/6411	1.05 (0.81–1.35)	1.01 (0.77–1.32)
Quartile 4	≥46.90	163/5739	1.26 (0.99–1.60)	1.19 (0.91–1.55)
*p* for trend			0.08	0.25

Multivariable Cox model was adjusted with age at diagnosis, race/ethnicity, education level, intervention group, menopausal status at baseline, total calorie intake, alcohol intake, smoking status, pack-years, physical activity, body mass index, tumor stage, tumor size, estrogen and progesterone receptor status, tamoxifen use, radiotherapy, and chemotherapy. Abbreviations: HR: hazard ratio; PRAL: potential renal acid load; NEAP: net endogenous acid production; WHEL: Women’s Healthy Eating and Living study; CI: confidence interval.

**Table 2:** Characteristics of breast cancer survivors at baseline by quartiles of the PRAL score (*n* = 3081).

	PRAL score quartiles (mEq/day)			
	Quartile 1	Quartile 2	Quartile 3	Quartile 4
	<−13.7 (*n* = 771)	−13.7 to <−3.7 (*n* = 769)	−3.7 to <4.7 (*n* = 771)	≥4.7 (*n* = 770)
**NEAL (mEq/day)^a^**	27.4 (23.9–30.8)	36.4 (33.7–38.5)	43.7 (41.1–46.3)	55.5 (50.9–61.5)
**Basic**				
Age at diagnosis (years)	52.0 (47.0–58.0)	51.0 (46.0–58.0)	50.0 (45.0–57.0)	48.0 (42.0–55.0)
White (%)	89.8	88.9	84.2	78.2
Normal weight (%)	56.7	46.7	36.7	32.6
Overweight and obese (%)	43.4	53.3	63.3	67.4
Education, at or above college (%)	63.7	55.7	51.9	45.8
Postmenopausal women (%)	84.3	80.6	79.2	73.0
Past smoker (%)	43.2	41.1	41.9	40.7
Never smoker (%)	53.7	54.5	53.2	53.8
Alcohol abstainer (%)	31.7	30.2	33.2	31.8
Physical activity (MET/week)	810 (300–1500)	615 (225–1305)	480 (150–1050)	398 (45–1080)
Chemotherapy (%)	65.9	68.7	71.7	73.4
Radiation (%)	63.6	61.0	59.1	62.2
ER+/PR+ (%)	63.3	63.5	62.4	58.2
ER-/PR- (%)	16.5	18.6	21.9	23.3
Cancer stage at diagnosis (%)				
I	39.2	37.3	38.3	39.5
II	55.1	59.2	57.1	54.4
IIIa	5.7	3.5	4.7	6.1
Tamoxifen use (%)	71.5	67.4	63.0	62.0
Hemoglobin A1c	5.60 (5.30–5.80)	5.60 (5.30–5.90)	5.60 (5.30–5.80)	5.60 (5.30–5.90)
**Nutrient intakes**				
Energy (KJ/day)	1667.0 (1455.0–1955.0)	1652.0 (1391.0–1902.0)	1638.0 (1387.0–1951.0)	1798.0 (1512.0–2124.0)
Carbohydrate (% of energy)	61.9 (56.9–67.1)	57.6 (52.4–61.9)	53.9 (49.1–59.0)	50.4 (45.4–53.7)
Fat (% of energy)	24.6 (20.4–29.0)	27.3 (23.3–31.8)	29.7 (25.3–34.5)	32.6 (28.1–36.7)
Protein (% of e nergy)	15.2 (13.2–17.1)	15.8 (13.9–18.2)	16.1 (14.1–18.4)	17.2 (15.1–19.7)
Calcium (mg/day)	821.0 (638.0–1104.0)	739.0 (567.0–967.0)	701.0 (534.0–901.0)	717.0 (550.0–920.0)
Phosphorus (mg/day)	1132.0 (934.0–1343.0)	1082.0 (878.0–1288.0)	1062.0 (867.0–1252.0)	1171.0 (959.0–1380.0)
Potassium (mg/day)	3559.0 (3090.0–4111.0)	2956.0 (2531.0–3392.0)	2584.0 (2198.0–3024.0)	2412.0 (2019.0–2932.0)
Magnesium (mg/day)	359.0 (299.0–420.0)	301.0 (256.0–355.0)	277.0 (233.0–330.0)	270.0 (218.0–324.0)

Abbreviations: PRAL: potential renal acid load; NEAP: net endogenous acid production; METS: metabolic equivalent/week; ER: estrogen receptor positive; PR: progesterone receptor positive. ^a^ Continuous variables are presented as median (inter-quartile range).

**Table 3:** Characteristics of breast cancer survivors at baseline by hemoglobin A1c (HbA1c) levels and the PRAL score.

	**HbA1**		**HbA1**	
	<5.6% (*n* = 1447)		≥5.6% (*n* = 1556)	
	PRAL score quartiles (mEq/day)		PRAL score quartiles (mEq/day)	
	Quartile 1	Quartile 4	Quartile 1	Quartile 4
	<−13.7 (*n* = 771)	≥4.7 (*n* = 770)	<−13.7 (*n* = 771)	≥4.7 (*n* = 770)
**NEAL (mEq/day) ^a^**	27.0 (23.3–30.3)	54.4 (50.4–60.6)	28.3 (24.5–31.4)	56.2 (51.4–61.7)
**Basic**				
Age at diagnosis (years)	50.0 (45.0–55.0)	46.0 (40.0–52.0)	54.0 (49.0–61.0)	50.0 (45.0–58.0)
White (%)	91.3	79.8	87.1	75.8
Normal weight (%)	62.8	41.7	45.8	19.1
Overweight and obese (%)	37.4	58.3	54.2	80.9
Education, at or above college (%)	64.1	52.6	62.9	35.8
Postmenopausal women (%)	79.4	66.5	92.7	82.6
Past smoker (%)	40.8	39.9	47.2	41.9
Never smoker (%)	56.3	55.3	49.3	51.6
Alcohol abstainer (%)	28.9	29.6	36.4	35.2
Physical activity (MET/week)	780 (300–1480)	500 (113–1200)	860 (315–1500)	255 (30–840)
Chemotherapy (%)	72.7	75.2	54.2	70.7
Radiation (%)	64.3	62.0	62.2	62.6
ER+/PR+ (%)	62.5	59.1	64.7	56.8
ER-/PR- (%)	18.0	23.0	13.6	23.6
Cancer stage at diagnosis (%)				
I	37.1	9.7	42.7	39.7
II	58.1	13.5	50.0	54.2
IIIa	4.7	1.5	7.3	6.1
Tamoxifen use (%)	65.3	60.2	78.7	62.3
Hemoglobin A1c	5.30 (5.10–5.40)	5.30 (5.10–5.40)	5.90 (5.80–6.00)	6.00 (5.80–6.40)
**Nutrient intakes**				
Energy (KJ/day)	1677.5 (1471.0–1979.5)	1811.0 (1527.0–2092.0)	1620.0 (1424.0–1920.0)	1796.0 (1486.0–2125.0)
Carbohydrate (% of energy)	62.9 (57.8–67.9)	51.1 (46.3–55.8)	60.1 (56.7–66.2)	48.7 (44.0–53.4)
Fat (% of energy)	24.2 (20.1–28.6)	31.1 (26.8–35.6)	25.1 (21.1–29.8)	34.2 (29.9–38.3)
Protein (% of e nergy)	14.7 (12.9–16.7)	17.1 (15.0–19.6)	15.7 (13.7–17.7)	17.3 (15.3–19.8)
Calcium (mg/day)	824.5 (630.0–1080.0)	727.0 (551.0–926.0)	817.5 (650.0–1114.0)	708.5 (528.0–889.0)
Phosphorus (mg/day)	1180.0 (930.5–1327.0)	1204.5 (956.0–1392.0)	1132.0 (924.0–1356.0)	1158.5 (962.0–1351.0)
Potassium (mg/day)	3531.5 (3093.5–4087.0)	2463.5 (2027.0–3008.0)	3254.0 (3053.0–4142.0)	2389.0 (2019.0–2863.0)
Magnesium (mg/day)	358.5 (301.5–423.5)	275.0 (221.0–337.0)	349.0 (294.0–413.0)	263.0 (218.0–309.0)

Abbreviations: PRAL: potential renal acid load; NEAP: net endogenous acid production; METS: metabolic equivalent/week; ER: estrogen receptor positive; PR: progesterone receptor positive. ^a^ Continuous variables are presented as median (inter-quartile range).

**Table 4:** Multivariable-adjusted associations between dietary acid load and breast cancer recurrence according to levels of hemoglobin A1c.

PRAL (mEq/day)	A1c < 5.3% (< 25%)		5.3% ≤ A1c < 5.6% (25%-< median)		5.6% ≤ A1c < 5.7% (median- <prediabetic)		A1c ≥ 5.7% (≥prediabetic range)	
Range	No. events/person-years	HR (95%CI)	No. events/person-years	HR (95%CI)	No. events/person-years	HR (95%CI)	No. events/person-years	HR (95%CI)
<−15.04	33/964	1	34/2038	1	8/448	1	23/1372	1
−15.04 to < −0.71	52/1615	0.80 (0.52–1.24)	77/3193	0.90(0.64–1.27)	17/640	2.87 (1.23–6.72)	53/2525	1.27 (0.80–2.02)
≥−0.71	49/1666	0.79 (0.51–1.22)	83/3148	0.76 (0.52–1.09)	16/729	2.48 (1.00–6.22)	72/2644	1.95 (1.27–3.00)
*p* for trend		0.31		0.14		0.05		0.001
*p* for interaction	0.01							
NEAP (mEq/day)	A1c < 5.3% (<25%)		5.3% ≤ A1c <5.6% (25%-<median)		5.6% ≤ A1c < 5.7% (median- <prediabetic)		A1c ≥ 5.7% (≥prediabetic range)	
Range	No. events/person-years	HR (95%CI)	No. events/person-years	HR (95%CI)	No. events/person-years	HR (95%CI)	No. events/person-years	HR (95%CI)
<31.5	36/994	1	34/2058	1	10/460	1	19/1372	1
31.5 to < 43.4	47/1599	0.84 (0.52–1.24)	80/3343	0.80(0.56–1.13)	15/638	1.28 (0.58–2.82)	58/2652	1.85 (1.16–2.95)
≥43.4	51/1652	0.86 (0.51–1.22)	80/2979	0.89 (0.62–1.27)	16/719	1.31(0.54–3.18)	71/2518	2.16 (1.36–3.42)
*p* for trend		0.56		0.16		0.35		0.001
*p* for interaction	0.05							

Multivariable Cox model was adjusted with age at diagnosis, menopausal status at baseline, total calorie intake, smoking status, pack-years, physical activity, body mass index, tumor size, and chemotherapy. Abbreviations: HR, hazard ratio; HbA1C, hemoglobin A1C; PRAL, potential renal acid load; NEAP, net endogenous acid production.

**Table 5:** Dietary acid load and risk of breast cancer recurrence, stratified by median levels of hemoglobin A1c in the WHEL cohort.

		A1c < 5.6%			A1c ≥ 5.6%		
PRAL (mEq/day)			HR (95%CI)			HR (95%CI)	
	Range	No. events/person-years	Age-adjusted	Mutivariable-adjusted	No. events/person-years	Age-adjusted	Mutivariable-adjusted
Quartile 1	<−19.50	44/2017	1	1	18/1132	1	1
Quartile 2	−19.50 to <–6.94	92/3349	0.91 (0.68-1.23)	0.88 (0.65–1.19)	47/2241	1.31 (0.83–2.08)	1.38 (0.85–2.17)
Quartile 3	−6.94 to <3.22	96/3742	0.90 (0.66–1.22)	1.12 (0.64–1.19)	57/2577	1.69 (1.08–2.62)	1.86 (1.19–2.93)
Quartile 4	≥3.22	96/3516	0.89 (0.66–1.22)	1.16 (0.59–1.13)	67/2409	1.93 (1.25–2.96)	2.15 (1.34–3.48)
*p* for trend			0.44	0.22		0.001	0.0005
*p* for interaction	0.01						
NEAP (mEq/day)							
	Range	No. events/person-years	Age-adjusted	Mutivariable-adjusted	No. events/person-years	Age-adjusted	Mutivariable-adjusted
Quartile 1	<28.44	51/2099	1	1	12/1134	1	1
Quartile 2	28.44 to <37.25	80/3099	0.98 (0.73–1.31)	0.96 (0.73–1.29)	51/2202	1.49 (0.93–2.37)	1.50 (0.95–2.44)
Quartile 3	37.25 to <46.90	99/3727	0.78 (0.57–1.09)	0.74 (0.53–1.02)	61/2684	1.84 (1.17–2.89)	1.97 (1.22–3.19)
Quartile 4	≥46.90	98/3400	0.99 (0.73–1.33)	0.92 (0.67–1.25)	65/2339	2.09 (1.34–3.23)	2.31 (1.42–3.74)
*p* for trend			0.71	0.39		0.0007	0.0004
*p* for interaction	0.05						

Multivariable Cox model was adjusted with age at diagnosis, race/ethnicity, education level, intervention group, menopausal status at baseline, total calorie intake, alcohol intake, smoking status, pack-years, physical activity, body mass index, tumor stage, tumor size, estrogen and progesterone receptor status, tamoxifen use, radiotherapy, and chemotherapy. Abbreviations: HR, hazard ratio; PRAL, potential renal acid load; NEAP, net endogenous acid production; WHEL, Women’s Healthy Eating and Living Study.

## Appendix A

**Table A1:** The associations of PRAL and PRAL-contributing foods with breast cancer recurrence in stratum with hemoglobin A1c ≥ 5.6%.

		**Estimates for PRAL**						**Estimates for each food**				
	Independent						Independent					
	variable	Quartile 1	Quartile 2	Quartile 3	Quartile 4	*p* for trend	variable	Quartile 1	Quartile 2	Quartile 3	Quartile 4	*p* for trend
		Ref	HR (95%CI)	HR (95%CI)	HR (95%CI)			Ref	HR (95%CI)	HR (95%CI)	HR (95%CI)	
Base model *	PRAL	Ref	1.38 (0.85–2.17)	1.86 (1.19–2.93)	2.15 (1.34–3.48)	0.0005	Fresh red meat	Ref	0.88 (0.51–1.54)	1.14 (0.66–1.96)	1.47 (0.87–2.47)	0.16
							Processed red meat	Ref	1.38 (0.75–2.54)	1.75 (0.98–3.13)	1.52 (0.84–2.75)	0.37
							Cruciferous vegetables	Ref	1.09 (0.71–1.69)	1.01 (0.63–1.61)	0.82 (0.47–1.36)	0.02
							Allium vegetables	Ref	1.51 (0.95–2.38)	1.93 (1.23–3.03)	0.78 (0.45–1.38)	0.34
							Other vegetables ^#^	Ref	0.84 (0.55–1.26)	0.63 (0.40–0.98)	0.82 (0.50–1.33)	0.27
							Legumes	Ref	1.04 (0.62–1.75)	0.69 (0.49–1.01)	0.64 (0.43–0.96)	0.01
							Soy legumes *	Ref	1.01 (0.65–1.55)	1.375(0.93–1.95)	NA	0.21
Mutually adjusted model ^	PRAL	Ref	1.41 (0.86–2.30)	1.83 (1.11–3.03)	1.96 (1.14–3.34)	0.0006	Fresh red meat	Ref	0.87 (0.50–1.51)	1.09 (0.64–1.88)	1.38 (0.82–2.32)	0.17
							Processed red meat	Ref	1.33 (0.72–2.45)	1.67 (0.94–2.99)	1.45 (0.80–2.63)	0.36
							Cruciferous vegetables	Ref	1.12 (0.72–1.72)	1.02 (0.64–1.62)	0.82 (0.48–1.40)	0.02
							Allium vegetables	Ref	1.44 (0.91–2.28)	1.90 (1.21–2.98)	0.77 (0.44–1.35)	0.27
							Other vegetables	Ref	0.88 (0.58–1.33)	0.68 (0.43–1.07)	0.98 (0.59–1.62)	0.87
							Legumes	Ref	1.09 (0.65–1.82)	0.71 (0.49–1.02)	0.66 (0.44–0.99)	0.02
							Soy legumes	Ref	0.99 (0.64–1.53)	1.37 (0.95–1.98)	NA	0.16

* Base model includes PRAL or 7 foods (7 foods were adjusted simultaneously) and other covariates in the base model; however, PRAL and 7 foods were not adjusted simultaneously. ^ Mutually adjusted model include both PRAL and 7 foods; PRAL and 7 foods were adjusted simultaneously. Covariates adjusted in base and mutually adjusted Cox models included: age at diagnosis, race/ethnicity, education level, intervention group, menopausal status at baseline, total calorie intake, alcohol intake, smoking status, pack-years, physical activity, body mass index, tumor stage, tumor size, estrogen and progesterone receptor status, tamoxifen use, radiotherapy, and chemotherapy. ^#^ Other vegetables include any vegetables except cruciferous, allium, and legumes. * Soy legume was classified as tertiles not quartiles due to a narrow range.
